# Intratympanic Injection of Dexamethasone and Electrocochleographic Data in Cases of Definite One Sided Refractory Meniere’s Disease

**Published:** 2017-05

**Authors:** Sasan Dabiri Satri, Reza Gharibi, Farzaneh Nejadian, Nasrin Yazdani, Reza Hoseinabadi, Nima Rezazadeh, Mohammad Reza Firouzifar, Saeed Babaei

**Affiliations:** 1 *Otorhinolaryngology Research Center, Tehran University of Medical Sciences, Tehran, Iran.*; 2 *Department of Audiology, School of Rehabilitation, Tehran University of Medical Sciences, Tehran, Iran.*; 3 *Department of Audiology, University of Social Welfare and Rehabilitation, Tehran, Iran.*

**Keywords:** Dexamethasone, EcochG, Meniere’s disease

## Abstract

**Introduction:**

Meniere’s disease (MD) is a disease of the inner ear that presents itself with repeated episodes of vertigo (96.2%), tinnitus (91.1%), and sensorineural hearing loss (87.7 %). In this article we sought to assess the effects of intratympanic (IT) injections of dexamethasone on definite cases of MD using electrocochleography (ECOG).

**Materials and Methods:**

In this hospital-based case series in 36 patients, we measured audiometric values and ECOG in all patients before, 1 month and 6 months after 4-mg/mL IT injections of dexamethasone.

**Results:**

Four patients (11%) had improved hearing following the intervention. No difference in pure tone audiometry (PTA) was observed following IT injections (P=0.492), while speech discrimination score (SDS) was significantly improved (P=0.008). There was a significant improvement in vertigo 1 month after IT injections (P<0.001), although this effect did not last for 6 months. No significant change in ECOG was observed between before and after treatment (P=0.052).

**Conclusion:**

IT dexamethasone injections can improve vertigo in definite cases of MD, although it seems that the effect is only temporary.

## Introduction

Meniere’s disease (MD) is a disease of the inner ear that presents itself with repeated episodes of vertigo (96.2%), tinnitus (91.1%), and sensorineural hearing loss (87.7 %). The prevalence of this disease is believed to be 3.5 to 513 in 100,000 people. The condition occurs in people of all ages, although it is more frequent in the fourth to the fifthdecade of life. The main pathogenesis is believed to be endolymphatic hydrops because of suboptimal absorption of endolymph by the endolymphatic sac (1).

There is no individual test for diagnosing MD. The American Association of Otolaryngology, Head and Neck Surgery (AAO-HNS) has developed criteria for diagnosing MD, which categorize the condition into four types: possible, probable, definite, and certain(1). In this article we studied definite MD, which is defined as two or more involuntary episodes of vertigo lasting for 20 or more minutes, and proven sensory neural hearing loss with audiometry, tinnitus, and fullness sensation.Electrocochleography (ECOG) is an electrophysiological test for recording potentials of the inner ear and cochlea in response to noise stimulation, consisting of three potentials: cochlear microphonic (CM), summating potential (SP), and action potential (AP) (1). CM is the intracellular action potential which shows basilar membrane movement; SP is the sum of action potentials created by hair cells; and AP is the sum of action potentials within the cochlear nerve. The presence of CM and SP highlights the normal state of hair cells.

ECOG is one method used to diagnose MD. Ferraro et al. assessed ECOG in the diagnosis of MD in a meta-analysis in 1999 and reported that the SP/AP ratio in a normal ear is within 0.16 to 0.31, compared with 0.4 to 0.5 in Meniere’s disease. Although normal ranges do not rule out the disease, a higher ratio is an important marker for endolymphatic hydrops (2).Although MD does not have a definite cure, a host of treatment options are available. Intratympanic (IT) injection of corticosteroids is one of the most debated treatment options in MD cases (3). Corticosteroids are believed to be effective in MD through their anti-inflammation effects, preserving the electrolyte balance within the cochlea and increasing the blood supply in the cochlea. This treatment is also easy to administer with local anesthesia and is economically favorable compared with surgical methods (4).

In this article, we sought to assess the effects of IT injections of dexamethasone in cases of definite MD using ECOG.

## Materials and Methods

This was a hospital-based case series in which we assessed cases of definite MD with regards to AAO-HNS criteria who presented at the Vertigo Clinic of Amir Alam Hospital, Tehran, Iran from March 2012 to March 2014. Patients were resistant to the maximal dose of medical therapy (salt limit, diuretic and betahistine) for at least 3 months. Inclusion criteria were 1) Age greater than 18 years; 2) No other otologic disorders; 3) Normal medical profile; 4) Normal magnetic resonance imaging (MRI) scan results; 5) No neurological diseases; and 6) Free will to participate. Exclusion criteria were 1) CNS abnormality; 2) Otitis media with or without effusion and conductive hearing loss; 3) Ototoxic drug consumption; 4) Opium addiction; 5) Past medical positive for surgical ear intervention; 6) Bilateral MD; 7) Tympanic membrane perforation; 8) Poor compliance to the study; 9) Systemic corticosteroid treatment within 1-month of injections.

Thirty-six patients were informed about the study, and asked to complete a form including name, age, sex, chief complaint (for example, hearing loss, vertigo, or tinnitus), disease duration, and signs. Then the patients underwent meticulous audiometric study, recording frequency, intensity and rate of hearing loss, SDS and speech reception threshold (SRT), acoustic reflex test, and tympanometry. Every patient underwent ECOG before IT injection of 4mg/mL of dexamethasone under local anesthesia in the anterior superior aspect of the tympanic membrane; an SP/AP ratio of more than 0.4 was considered positive.

Patients were told to remain in the supine position for 5 minutes after the injection and to avoid swallowing during that time. Three injections were carried out within a 1-week interval. Four weeks after the last injection, ECOG and audiometry tests were repeated. Patients were followed for 1 and 6 months after the injections.We assessed improvements in vertigo using a numeric value based on AAO-HNS criteria (Table.1). Hearing improvements, if any, were recorded regarding the qualitative measures of AAO-HNS and quantitatively using PTA and SDS.

**Table 1:** Numeric scale value for vertigo based on AAO-HNS

Numeric value	Control level	Class
0–40	Complete control of definitive spells	A
41–80	Limited control of definitive spells	B
81–120	Insignificant control of definitive spells	C

Tinnitus was recorded subjectively as absent, better, worse, or the same. Data were analyzed using descriptive analysis. Vertigo was analyzed using a nonparametric Friedman test then Wilcoxon signed ranks. Hearing was evaluated on both qualitative (descriptive) and quantitative measures (Wilcoxon signed ranks for PTA, SDS and ECOG). Tinnitus was analyzed using the McNemar test.Our study design was approved by the Ethics Committee of Amir Alam Hospital. All patients were deliberately included and were free to leave the study at will. All expenses were supported by the Center for Ear, Nose, and Throat Studies at Amir Alam Hospital.

## Results

We evaluated 36 cases of unilateral, drug resistant, definite MD who presented to the Amir Alam Hospital, Tehran, Iran from 2012 to 2013. Data were collected regarding patients’ age, sex, family history, duration of disease, hearing level (PTA, SDS), and frequency and duration of vertigo. Patients underwent IT injections of 4 mg/mL dexamethasone. Before and 4 weeks after the injections, ECOG was recorded. The intensity and frequency of vertigo was followed, as well as hearing level and tinnitus, 1 and 6 months after the injections.

The mean ± standard deviation (SD) age was 39.89±13.86 years, with the youngest and oldest patients being 20 and 72 years old, respectively. Twenty-one male (58.3 %) and 15 female (41.7%) patients were included. The mean ± SD duration of disease was 4.06±3.11 years, with a minimum and maximum duration of 1 and 15 years, respectively. Seven (19.4%) and 29 patients (80.6%) had a positive and negative family history for the disease, respectively.

**Fig 1:**
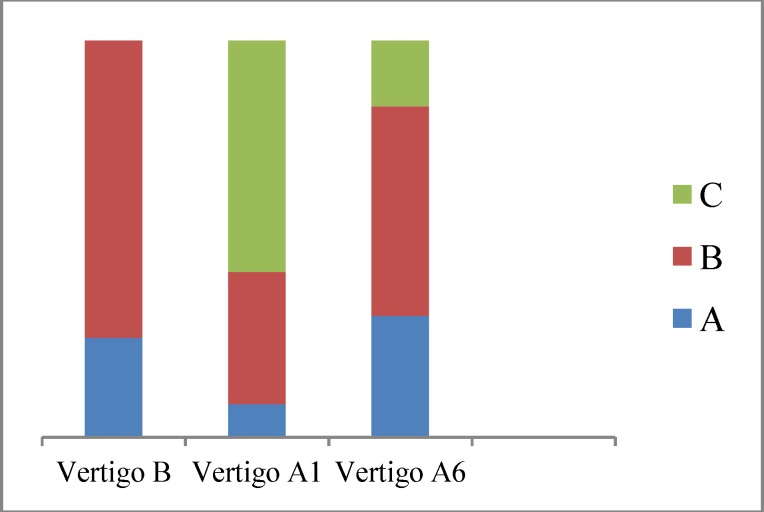
Numeric scale value of vertigo before IT injection, one month and 6 months after injection Vertigo B: degree of vertigo before injection; Vertigo A1: degree of vertigo 1 month after injection, Vertigo A6: degree of vertigo 6 months after injection

The numeric scale value of vertigo before the IT injections, and 1 month and 6 months after injection is shown in Table 2 and Fig 1.

**Table 2:** Distribution of vertigo classes (A, B, or C) based on numeric scale value for vertigo

	**C**	**B**	**A**
Vertigo B	0	27	9
Vertigo A1	21	12	3
Vertigo A6	6	19	11

We used Friedman and Wilcoxon signed ranks test to analyze these three vertigo classes based on different numeric scales. There was a statistically significant difference between degree of vertigo before injection and 1 month after the injections (P<0.001) and between 1 and 6 months after the injections (P<0.001); however, there was no statistically significant difference in vertigo before and 6 months after the injections (P=0.2). Hearing level was measured both qualitatively and quantitatively. A qualitative assessment of hearing was based on AAO-HNS criteria (Table.3). Based on the AAO-HNS criteria, 20 patients (55.6%) were in stage 1, 11 (30.6%) were in stage 2 and five (13.9%) were in stage 3 before dexamethasone treatment. After treatment, four patients (11%) improved in hearing level while the remaining 32 patients (88%) were unchanged. No patients experienced worsening of hearing during our study.For the quantitative hearing level assessment, we measured PTA and SDS in all patients. 

**Table 3:** AAO-HNS criteria for hearing level assessment

**Stage**	**Four-Tone Average (dB)**
1	≤25
2	26-40
3	41-70
4	>70

a. Hearing is measured using a four-frequency pure-tone average (PTA) of 500HZ, 1KHZ, 2KHZ, and 3KHZ

b. Pretreatment hearing level: worst hearing level during 6 months before surgery

c. Post-treatment hearing level: Poorest hearing level measured 18-24 months after institution of therapy

d. Hearing classification:

i. Unchanged: ≤10-dB PTA improvement or worsening or ≤15% speech discrimination improvement or worsening

ii. Improved: >10-dB PTA improvement or >15% discrimination improvement

iii. Worse: >10-dB PTA worsening or >15% discrimination worsening

In 1996, the Committee on Hearing and Equilibrium reaffirmed and clarified the guidelines, adding initial staging and reporting guidelines:

Mean±SD PTA decrement level was 20.86±14.55 dB before and 19.94±15.16 dB after injection; with no significant difference (P=0.492). Mean ± SD SDS was 92.56±8.477 before and 94±8.15 after injection. The Wilcoxon signed ranks test analysis showed a significant difference between these two values (P=0.008).ECOG results are shown in Table 4. There was no statistically significant difference in ECOG before and after the intervention (P=0.052).

Among the 36 patients, 32 had tinnitus (88.9%) and four were tinnitus free (11.1%) before the intervention. After the injections, 28 patients (77.8%) had tinnitus and eight patients (22.2%) were tinnitus free; this difference was not statistically significant according to the McNemar test (P=0.344).

**Table 4:** ECOG results before and after injection

**ECOG Results**		**Frequency**		**Percent**	
		Before	After	Before	After
Valid	Positive	19	11	52.8	30.6
	Negative	14	23	38.9	63.9
	Undiagnostic	3	2	8.3	5.6
	Total	36	36	100.0	100.0

## Discussion

This is the first study to assess response to IT dexamethasone in drug-resistant MD using ECOG in Iran.

One month after IT dexamethasone injections, 75% of patients saw an improvement of at least one class using a numeric value vertigo scale. After 6 months, the vertigo scale fell dramatically to 19.5%, which could be a sign of the short-term benefit of this intervention.

Assessment of vertigo before and 1 month after the injections using AAO-HNS criteria demonstrated a significant difference between the two time points (P<0.001). The same result was also seen between 1 month and 6 months after the injections (P<0.001). There was no significant difference between the results before and 6 months after injections (P=0.2), which could suggest a short-term effect of the intervention or could be because of the low power of this study, case limitation and a small sample size. This result was in agreement with the findings of Martin-Sanz et al. who evaluated the changes in ECOG after IT dexamethasone injections. They found a significant transient improvement in vertigo control which did not persist after 1 year of follow up (5). These results were comparable with a study by Barrs et al. in 1999–2004 in 21 drug-resistant cases of MD. They reported an improvement in vertigo 3 and 6 months after IT dexamethasone of 52% and 43%, respectively (6,7).

In contrast, our results are not consistent with those of several other studies. In another study by Martin-Sanz et al., for example, ECOG was evaluated in order to monitor the response of IT steroid therapy in MD patients refractory to medical therapy. Complete control of vertigo was achieved in 41.5% of patients after 12 months follow up and 15.1% after 24 months of follow up. In this study, IT injections of dexamethasone were performed every week for 3 consecutive weeks (8). Silverstein et al. performed a double-blind randomized trial in 20 cases of definite and possible MD in 1998. After three injections they reported that IT injection of dexamethasone has no significant effect compared with placebo (3).

Our results indicate that there is no correlation between hearing and tinnitus in patients, although several previous studies have indicated the presence of such a correlation. For example, Herraiz et al. reported that transtympanic methylprednisolone has long-lasting effects on improving all three of the main signs of MD (9).

ECOG sensitivity in our study was 52.8 %. Bearing in mind that our cases were definite MD, the sensitivity we obtained was lower than in other studies. Martin-Sanz et al. assessed the sensitivity of ECOG in 100 MD cases, among which 62 were definite, 13 were probable, and 25 were possible. 

A sensitivity of SP/AP of greater than 0.5 was reported in 92%, 78% and 75%, respectively, for each type of MD (10). Daneshi et al. also obtained 67% sensitivity for ECOG in a sample of 100 Iranian patients (11).

ECOG before and after IT injection of dexamethasone were not significantly different, although there was a trend toward difference (P=0.052). This could be because of the low power of the study, which was in turn was due to the small sample size. Therefore, this result could suffer from a type-B error. In our study, more males than females were included, while in epidemiologic studies, MD is more common in females (1).

## Conclusion

Based on our results and other previous results, IT injections of dexamethasone can alleviate vertigo, as the most common and serious symptom of MD, for at least a 3-month period, and ablative methods can be prevented. However, this effect does not persist for long and repeated medication is mandatory.
